# GPOPSIM: a simulation tool for whole-genome genetic data

**DOI:** 10.1186/s12863-015-0173-4

**Published:** 2015-02-05

**Authors:** Zhe Zhang, Xiujin Li, Xiangdong Ding, Jiaqi Li, Qin Zhang

**Affiliations:** Guangdong Provincial Key Lab of Agro-Animal Genomics and Molecular Breeding, College of Animal Science, South China Agricultural University, Guangzhou, 510642 China; Key Laboratory of Animal Genetics and Breeding of the Ministry of Agriculture, National Engineering Laboratory for Animal Breeding, College of Animal Science and Technology, College of Animal Science and Technology, China Agricultural University, Beijing, 100193 China

**Keywords:** Data simulation, SNP, Pedigree, Multiple traits, Mutation-drift equilibrium, Genetic correlation

## Abstract

**Background:**

Population-wide genotypic and phenotypic data is frequently used to predict the disease risk or genetic/phenotypic values, or to localize genetic variations responsible for complex traits. GPOPSIM is a simulation tool for pedigree, phenotypes, and genomic data, with a variety of population and genome structures and trait genetic architectures. It provides flexible parameter settings for a wide discipline of users, especially can simulate multiple genetically correlated traits with desired genetic parameters and underlying genetic architectures.

**Results:**

The model implemented in GPOPSIM is presented, and the code has been made freely available to the community. Data simulated by GPOPSIM is a good mimic to the real data in terms of genome structure and trait underlying genetic architecture.

**Conclusions:**

GPOPSIM would be a useful tool for the methodological and theoretical studies in the population and quantitative genetics and breeding.

**Electronic supplementary material:**

The online version of this article (doi:10.1186/s12863-015-0173-4) contains supplementary material, which is available to authorized users.

## Background

Single nuclear polymorphism (SNP) markers are widely implemented in the investigation of human genetics and animal/plant breeding, due to its high abundance and extensive coverage across the whole-genome. They were usually used to predict the disease risk in human [1,2], to localize genetic variations responsible for complex traits through genome wide association study (GWAS) [3], and to predict the genetic values of economically important traits in plant and animal breeding [4,5]. The techniques and methodologies related to this discipline are moving fast, and these new methods need to be evaluated before implemented to real data. The most efficient way for such kind evaluation is computer simulation.

Data simulation has been employed in genetic analysis for decades. Recently, many novel findings in genomic prediction using simulated whole-genome data were reported [6,7]. The most commonly used model for whole-genome genotypic data simulation is the mutation-drift equilibrium (MDE) model [8]. However, the rules applied in the MDE model vary in different studies, which made results from different studies incomparable. Meanwhile, only independent traits could be simulated by most programs, and function of simulating multiple correlated traits are seldom to be developed.

We present GPOPSIM: a simulation tool for population genetic data based on MDE. The mechanism to create polymorphic markers, population structure, and trait phenotypes were detailedly proposed. Moreover, simulating multiple genetically correlated traits were explored as well. In order to demonstrate the performance of our program, a series of implementation were carried out in this study.

## Implementation

In this section, we describe the implementation of the method from [9] in the presented software GPOPSIM. The software can be compiled and executed in multiple platforms, and driven by a parameter file. The parameter setting is illustrated in Table 1 and more details could be found in the project home page (https://github.com/SCAU-AnimalGenetics/GPOPSIM).

**Table 1:** **Parameter setting**

**Category**	**Parameters**
Overall	population stages, number of sub populations in the current population stages, chromosome number, chromosome length
Marker	marker number per chromosome, marker distribution, mutation rate for marker& QTL
QTL	QTL effect distribution, QTL number, QTL ratio for multiple trait simulation
Trait	trait number, trait type, heritability, correlations between traits
Population setting	population size, number of sires selected, number of dams selected, number of generations, selection rule, matting rule, mutation rule

**Figure 1:**
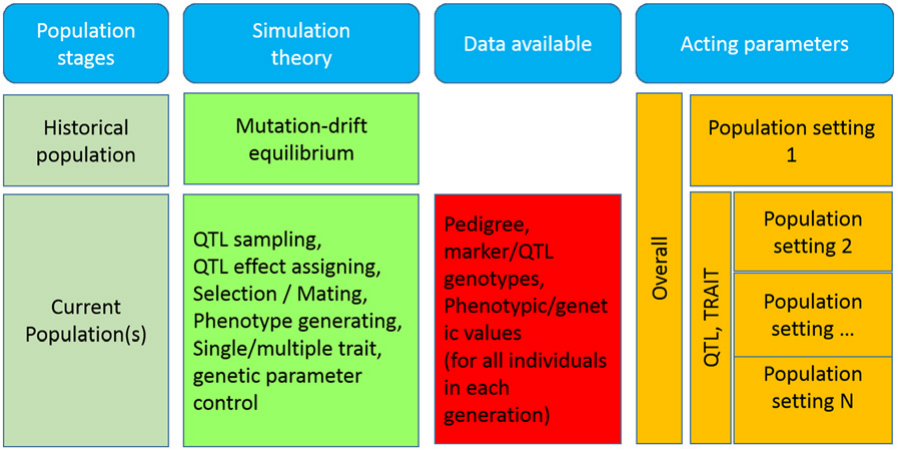
**Workflow and parameter setting in GPOPSIM.**

The simulation of whole-genome genotypes is based on the MDE model [8]. It starts from an initial population, through many generations of historical population, ends to the current population. In this process, the polymorphism of markers is increased by mutation, but decreased by genetic drift, and reaches equilibrium status throughout a number of historical population, which was named mutation-drift equilibrium [10]. The whole-genome data generated in the current population can be used for data analysis. Figure 1 illustrates the workflow and acting parameter categories in each population stage.

### Population structure

The populations simulated by GPOPSIM include one historical population and one or more current population(s). The population structures can be very flexible in different population stages by assigning parameters such as different population sizes, number of selected breeding male and female, the selection rules and other parameters for each population stage (Table 1, Figure 1). The population/pedigree structure of the simulated data is decided by the parameter settings of the current populations. The parameter settings for the historical population mainly affect the genome structure of the current population.

### Genome structure

The genome structure could be clearly defined with the overall parameters and mutation rules applied in each current population. Generally, the number of chromosome and the lengths of different chromosomes are arbitrarily assigned [4,11,12], e.g. 1 Morgan for each of five chromosomes. The number of markers on each chromosome could vary, and each segment between two adjacent markers was considered to harbor a potential QTL. In GPOPSIM, the position of markers and potential QTLs were simply assumed a mixture of uniform and exponential distribution to mimic the real SNP data in currently available SNP chips [9], such as the Illumina BovineSNP50 BeadChip [13].

The polymorphic markers and the linkage disequilibrium (LD) among them are mainly created in the historical population. The expected marker heterozygosity (*H*_*e*_) is *H*_*e*_ = (4*N*_*e*_*u*)(4*N*_*e*_*u* + 1)^−1^ [10], where *N*_*e*_ is the effective population size and *u* is the mutation rate. And the expected LD is *r*^2^ ≈ 1/(α + k*N*_*e*_*c*) [8], where α is an indicator of mutation, *c* is the genetic distance between markers.

### Genetic and phenotypic values

Based on the genome structure generated in the historical population, the trait and QTL parameters, GPOPSIM simulates genetic and phenotypic values for each individual in the current population. The true QTLs are randomly sampled from all candidate QTLs. The true genetic effects of each true QTL are sampled from normal [1] or gamma distribution [4]. By setting different QTL number and effect distribution, a wide range of genetic architecture from simple disease traits to complex traits can be simulated easily. For each trait, the true genetic merit of one individual is defined as the cumulative effect across all true QTLs. For quantitative traits, the phenotypic value is generated by adding the true genetic merit with a random residual error, while the 0/1 phenotype is generated by setting an incidence for threshold traits.

The principles applied to single-trait data simulation can be easily extended to two or multiple genetically correlated traits simulation. For two traits simulation, more flexible parameters and rules can be applied. All true QTLs affecting both traits are divided into three groups: (1) Group1 is a group of true QTLs simultaneously affecting both traits, in which their effects are sampled from a multivariate normal distribution or a gamma distribution [14], (2) the true QTLs in Group2 and Group3 affect only one of the two traits, respectively, for which the effect of each causative locus in Group2 and Group3 is sampled from a normal or gamma distribution. The genetic correlation between two traits ranged from −0.88 to 0.88, which can basically cover the genetic correlated traits. Random residual errors are sampled from a multivariate normal distribution. Similarly, the phenotypic value and genetic merit of one individual on both traits are generated as the single trait module does. Considering the sampling error of simulation, the expected genetic correlation (*r*_g_) of two traits is evaluated and provided by GPOPSIM according to the formula [15]

1 $$ {r}_{AB}={\displaystyle \sum 2{p}_i\left(1-{p}_i\right){\alpha}_{Ai}{\alpha}_{Bi}}/\left(\sqrt{{\displaystyle \sum 2{p}_i\left(1-{p}_i\right){\alpha}_{Ai}^2}}\ast \sqrt{{\displaystyle \sum 2{p}_i\left(1-{p}_i\right){\alpha}_{Bi}^2}}\right) $$

where *p*_*i*_ is the frequency of one of two alleles for the locus *i*; *α*_*Ai*_ is the effect of the locus *i* for Trait A; *α*_*Bi*_ is the effect of the locus *i* for Trait B. For multiple traits simulation, all true QTLs are assumed to affect all traits simultaneously for simplicity and their effects are sampled from a multivariate normal distribution with the restriction of assigned genetic correlations [16].

### Input and output files

Only one input file, also being the parameter file is needed to run GPOPSIM (Table 1). Generally, GPOPSIM generates four types of output files: (1) a data file including pedigree information, the individuals and their parents identities, and the simulated true genetic value and phenotypic value for each trait and each individual; (2) marker genotype file providing the marker genotypes in phased format; (3) QTL genotype file providing the true QTL genotypes; and (4) several separate parameter files include a marker map file, a true QTL map file including their simulated true QTL effects, and a genetic parameters file. All these files are in text format with the file extension of ‘.txt’. And the first three types of files are generated for each generation with a filename including the number of generation.

### Source code and software availability

Based on the methods described above and in [9], we developed a whole-genome data simulation software GPOPSIM in Fortran 90 and tested on Microsoft Windows (version XP/7/8), and Linux (Red Hat Enterprise, Ubuntu, Fedora). It can simulate population with various population structure, genomic data, one or more independent/correlated continuous trait(s). The volume of simulated dataset depends on the running environment of the user’s PC or server.

A series of simulations were carried out to investigate the quality of the simulated data using GPOPSIM, and the Haploview software [17] was used for data quality control and linkage disequilibrium analysis. The variance components and genetic correlations were estimated by DMU [18].

## Results and discussion

We describe the quality of data simulated by GPOPSIM first, and followed by a general discussion of the implementation of GPOPSIM.

**Figure 2:**
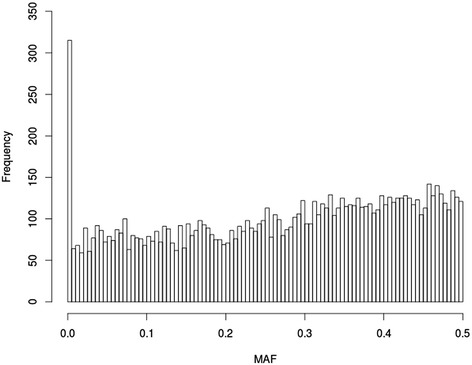
**Distribution of the minor allele frequency (MAF) of genotypes simulated by GPOPSIM.** Parameter setting for this simulation is *Ne* = 100, mutation rate *u* = 2.5 × 10^−3^, number of markers = 10,000.

Besides the features predefined by users, e.g. marker density, minor allele frequency (MAF) and LD can typically reflect the characteristic of the simulated genotypic data. Usually, MAF in the current population generated by GPOPSIM nearly follows an uniform distribution with a long tail near MAF = 0, which is also called “L” shape distribution of MAF, or “U” shape distribution on the entire frequency spectrum. Figure 2 shows the distribution of MAF in the scenario with *N*_*e*_ = 100 and *u* = 2.5 × 10^−3^, nearly 50% loci’s MAF were lower than 0.3, and the average MAF was 0.28, which is similar to the average MAF in Holstein detected with Illumina Bovine50SNP BeadChip [13,19]. The average MAF and heterozygosity could be altered by increasing or decreasing the value of mutation rate *u* in the historical population [9].

**Figure 3:**
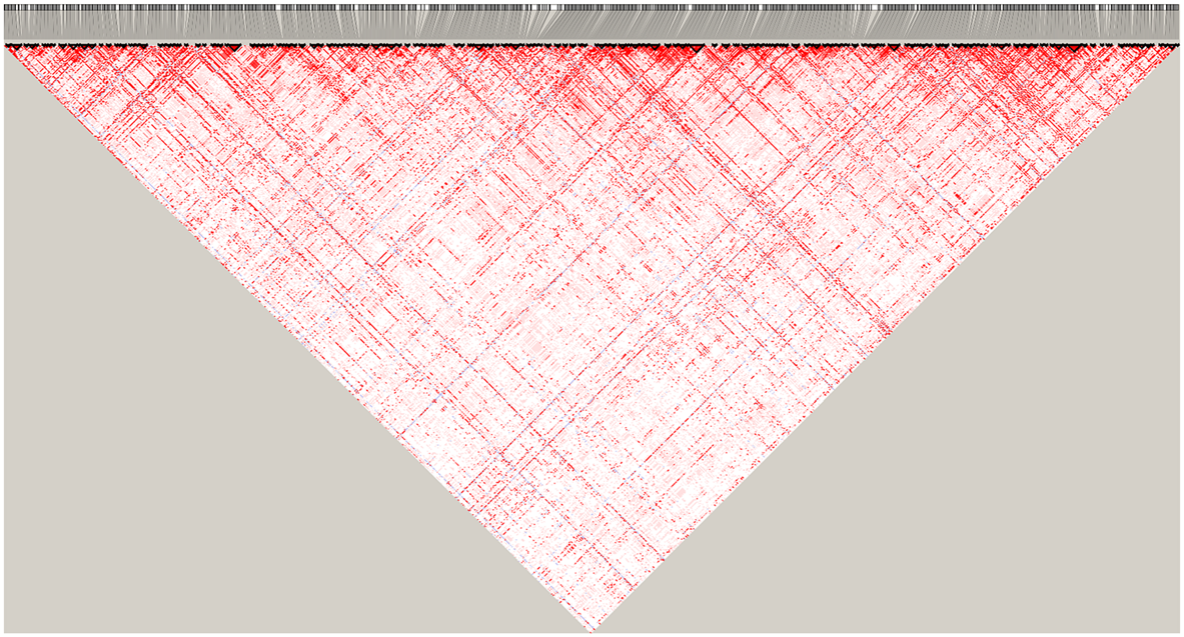
**Pattern of linkage disequilibrium (LD) of the genotypes simulated by GPOPSIM.** Parameter setting for this simulation is *Ne* = 100, mutation rate *u* = 2.5 × 10^−3^, number of markers = 10,000. The pairwise LD among the first 1000 markers were shown in this figure.

Linkage disequilibrium is another indicator for the quality of simulated genotypic data. Figure 3 illustrates the LD pattern of simulated data in the same scenario as in Figure 2, the average LD between adjacent markers is 0.24. High LD can be observed in both long range and short range (Figure 3), additionally, haplotype blocks can be found as well, these fit the real data very well [19].

We assessed the two-trait phenotypic data simulated by GPOPSIM by comparing the assigned and estimated genetic parameters on condition that partial common QTLs affect both traits. We set two genetically correlated traits (denoted as Trait A and Trait B) with heritability of 0.1 and 0.3, the genetic correlation between trait A and B was assigned 0, 0.2, 0.5 and 0.8, and the environmental correlation was set 0. From the results of 20 replicates of simulation (10,000 individuals in each replicate) (Table 2), we can see that the heritability estimated by DMU are very close to the assigned values in different levels of genetic correlations and the estimation vary in a very small range among replicates. Likewise, the estimations of genetic correlation from DMU are acceptable and close to those assigned, in addition, these estimations are also nearly same as those expected genetic correlations, which are calculated from equation 1. This indicates that GPOPSIM can be an ideal tool for simulating multiple traits with/without genetic correlation. The bias with the preset genetic correlations is acceptable. Besides, the estimates of variance components at all levels of genetic correlation fit the assigned values very well (Table 2).

**Table 2:** **The assigned and estimated heritability (h** ^**2**^ **), genetic correlation (r**_**g**_ **) and residual correlation (r**_**e**_ **) for two trait phenotypes simulated by GPOPSIM**

***h*** ^**2**^		***r***_**g**_			***r***_**e**_
**Estimated A**	**Estimated B**	**Assigned**	**Expected**	**Estimated**	**Estimated**
0.101(0.011)	0.289(0.022)	0.0	0.000(0.000)	−0.004(0.092)	0.003(0.016)
0.100(0.009)	0.302(0.025)	0.2	0.180(0.046)	0.159(0.103)	−0.002(0.010)
0.100(0.012)	0.299(0.022)	0.5	0.506(0.045)	0.493(0.079)	0.004(0.011)
0.104(0.012)	0.290(0.024)	0.8	0.805(0.042)	0.805(0.078)	0.000(0.015)

The assigned heritability is 0.1 and 0.3 for trait A and B, respectively; the assigned residual correlation is 0; the mean (S.D.) of estimated genetic parameters were obtained from DMU and calculated from 20 replicate of simulations.

GPOPSIM is distributed both as Fortran 90 source code and as executable procedure on Windows and Linux platform (https://github.com/SCAU-AnimalGenetics/GPOPSIM or Additional file 1). It is free of charge for all purpose users and no license is required. The computing time and RAM demanding on PC, with 3.0 GHz CPU, 2 GB RAM is 4.4 minutes and 8 Mb, respectively, for simulating 10000 markers, 1000 historical generations with *Ne* = 100. The time demanding increased nearly linearly with the effective population size *N*_*e*_, number of markers *N*_*m*_ and number of generations *N*_*g*_.

GPOPSIM is designed for, but not limited to, data simulation in genetic or breeding researches that needs genomic and phenotypic data from a population, such as genome wide association study, whole genome prediction, population genomics studies, and genomic selection breeding program. Though GPOPSIM has been successfully implemented in our previous studies [11,20,21], there is still rooms for further extension, such as sequences data simulation.

## Conclusions

We presented GPOPSIM, a simulation tool for pedigree, phenotypes, and genomic data, with a variety of population and genome structures and trait genetic architectures. It enables users to simulate (1) various genome structures via mutation drift equilibrium model with user defined historical population parameters; (2) pedigree from one or more current population(s) with flexible user assigned population structure parameters; (3) phenotypes on single or multiple traits with/without desired genetic correlation and genetic architectures. GPOPSIM is designed for, but not limited to, data simulation in genetic or breeding researches that needs genomic and phenotypic data from a population, such as genome wide association study, whole genome prediction, population genomics studies, and genomic selection breeding program. The software can run on multiple platforms and the code has been made freely available to the community. We speculated that this software could promote the methodological and theoretical studies in the discipline of population and quantitative genetics and breeding.

## Availability and requirements

**Project name:** GPOPSIM

**Project home page:**https://github.com/SCAU-AnimalGenetics/GPOPSIM

**Operating system(s):** Compiled for Windows and Linux

**Programming language:** Fortran 90

**Other requirements:** None

**License:** None

**Any restrictions to use by non-academics:** None

## Additional file

Additional file 1:
**A compressed file includes the GPOPSIM resource code (GPOPSIM.f90) and the parameter file for GPOPSIM (para.txt).**

## Abbreviations

GPOPSIM: Genome-wide population simulation
SNP: Single nuclear polymorphism
GWAS: Genome wide association study
GS: Genomic selection
MDE: Mutation-drift equilibrium
MAF: Minor allele frequency
LD: Linkage disequilibrium
